# Morbidity in open versus minimally invasive hybrid esophagectomy (MIOMIE): Long-term results of a randomized controlled clinical study

**DOI:** 10.1007/s10353-018-0552-y

**Published:** 2018-08-07

**Authors:** Matthias Paireder, Reza Asari, Ivan Kristo, Erwin Rieder, Johannes Zacherl, Barbara Kabon, Edith Fleischmann, Sebastian F. Schoppmann

**Affiliations:** 1 Department of Surgery, Upper-GI-Service Comprehensive Cancer Center, GET-Unit Medical, University of Vienna, Spitalgasse 23, 1090 Vienna, Austria; 2 Department of Surgery, St. Josef Hospital of Vienna, Vienna, Austria; 3 Department of Anesthesia, Medical University of Vienna, Vienna, Austria

**Keywords:** Minimally invasive surgical procedures, Esophageal cancer, Esophageal resection, Laparoscopy

## Abstract

**Background:**

The minimally invasive esophagectomy (MIE) for esophageal cancer was introduced assuming a reduction of morbidity and operation time. After implementation of MIE at our institution, a randomized controlled trial was designed.

**Methods:**

This is a prospective randomized controlled study comparing open (OE) and laparoscopic gastric tube (MIE) formation in Ivor Lewis esophagectomy. Primary endpoints were morbidity and 30-day mortality. Secondary endpoints included the duration of intensive care unit stay, length of hospital stay, operative time as well as relapse-free and overall survival.

**Results:**

Twenty patients (76.9%) were male, median age was 63 years (40–77). Median operation time was 290 (215–385) minutes in OE and 292.5 (200–450) minutes in MIE group, *p* = 0.421. Major complications occurred in 4 (33.3%) patients in the OE group and in 6 (35.7%) patients in the MIE group. Anastomotic leakage was seen in 2 (16.6%) and 3 (21.4%) patients, respectively (OR 1.364; CI = 0.188–9.912; *p* = 0.759). Due to an alarming number of consecutive anastomotic leakages, the trial was stopped after inclusion of 26 patients. Median follow-up was 41.5 (1–62.6) months. 5‑year survival rate was 50%. Thirty-eight percent developed recurrence of disease in the study period. There was no significant difference in overall and relapse-free survival regarding the type of surgery.

**Conclusion:**

This study shows that hybrid MIE is a feasible alternative for esophageal resection. Morbidity, mortality, and oncological long-term results were equal in both groups, but the interpretation has to be done carefully due to premature termination of the trial. Interrupting a trial because of patient benefit should not be a reason to discard results but rather to improve technical aspects and strive for novel studies.

## Main novel aspects

- This study presents long-term outcomes after minimally invasive esophagectomy.
- Minimally invasive esophagectomy does not jeopardize oncological outcome.
- Morbidity and mortality are not increased in minimally invasive esophagectomy.

## Introduction

Establishment of multimodal treatment concepts for patients with advanced esophageal cancer has improved outcomes over the last few years [1]. Though surgery remains the only option for long-term survival in patients with localized cancer stage, esophageal resections are associated with considerable morbidity and mortality [2]. Standardized perioperative efforts could improve the outcome of these patients [3] While early reports of medical pioneers focused particularly on safety and feasibility, more recent studies showed that implementation of minimally invasive esophagectomy (MIE) was widely accepted [4,5,6,7]. Since first reports of MIE, different techniques and adjustments have been discussed. A recent publication of a large prospective trial in phase II showed the safety of a total minimally invasive approach (video-assisted thoracoscopic surgery [VATS] and laparoscopy). However, surgical technique is still a subject of debate and the level of evidence remains low [8]. Proving feasibility does not warrant a paradigm shift, as experience is an important factor for safety and patient benefit.

The aim of this study was to evaluate morbidity and long-term results of open esophagectomy (OE) versus hybrid MIE in a randomized controlled setting.

## Materials and methods

### Study protocol

The MIOMIE trial is a prospective randomized controlled trial (RCT) comparing OE (open gastric mobilization and right thoracotomy with intrathoracic anastomosis) with hybrid MIE (laparoscopic gastric mobilization and open right thoracotomy and intrathoracic anastomosis). Primary endpoints were morbidity (anastomotic leakage, gastric conduit necrosis, or pneumonia) and 30-day mortality. Secondary endpoints included duration of intensive care unit (ICU) stay, length of hospital stay, and operative time as well as long-term outcomes, such as relapse free survival (RFS) and overall survival (OS).

Patients diagnosed with esophageal cancer were routinely staged with esophagoscopy and computed tomography (CT) or positron emission tomography/computed tomography (PET/CT). In cases of locally advanced tumor stage or lymph node positivity, neoadjuvant treatment was initiated. After re-staging, if patients were found eligible for Ivor Lewis esophagectomy, randomization was performed prior to surgery. Patient recruitment started in April 2010 and follow-up was performed until April 18, 2016. Informed consent was obtained from all patients for being included in the study. The local institutional review board approved the study protocol. The trial was registered before publication at clinicaltrials.com (NCT03035071).

### Randomization

**Fig. 1:**
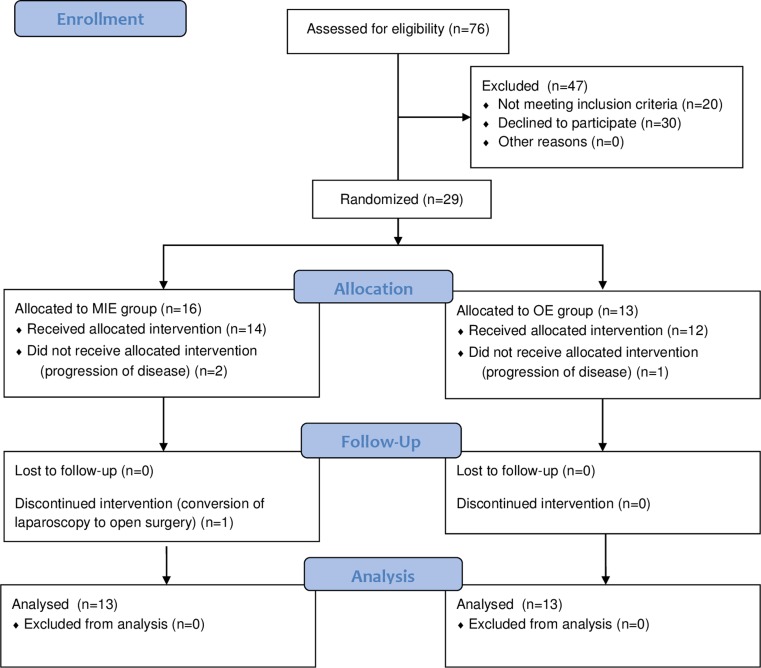
Flow chart depicting the patient selection and randomization process. *MIE* minimally invasive esophagectomy, *OE* open esophagectomy

A computer-based online randomizing tool, provided by the Medical University of Vienna, was used to perform randomization in the evening before day of surgery [9]. Patients were either randomized to open surgery or to minimally invasive laparoscopic gastric tube formation. Randomization was performed by the study center. Please find the CONSORT flowchart in Fig. 1.

### Inclusion criteria

This study included patients with adenocarcinoma (AC) of the esophagus and the esophagogastric junction in Siewert I and II position as well as esophageal squamous cell cancer (ESCC) who required abdominothoracic esophageal resection [10]. Patients between 18 and 80 years, who gave their informed consent prior to randomization, were eligible for this study.

### Exclusion criteria

Individuals who did not meet the inclusion criteria were excluded from the study. Patients with tumor localization in the upper third of the esophagus and requiring cervical resection were excluded. Patients presenting other than AC or ESCC or showing a contraindication for laparoscopy (history of large abdominal surgery or signs of hostile abdomen) were excluded from this study. Also, patients with a history or presence of any other malignancy, except carcinoma in situ or basalioma, were not eligible for this study.

### Surgery

In all patients, abdominothoracic resection with right anterolateral thoracotomy was performed. Standard en-bloc two-field lymphadenectomy was conducted as described by Jamieson et al. [11]. In the OE group, a transverse upper abdominal laparotomy was used as access for gastric mobilization. In the MIE group, the laparoscopic procedure was performed as was extensively described elsewhere [12]. In brief, the patient was placed in supine position with legs apart. The surgeon stands between the legs using a five-trocar technique. Esophagogastric anastomosis was performed using a circular stapling device (CDH-25, Ethicon US, LLC). Esophageal mucosa was secured with interrupted PDS 4/0 (Ethicon US, LLC) sutures prior to anastomosis. Drainage placement (one pleural left-thoracic, one pleural right-thoracic in ventral position, one right-thoracic in dorsal position nearby the anastomosis) was conducted similarly in both groups.

### Survival and morbidity

Overall survival and relapse-free survival were defined as the period from the operation until death or recurrence of disease, respectively. Morbidity was grouped in Clavien/Dindo (C/D) classification [13]. C/D grades I & II were considered as minor complications, III a,b and IV a,b were referred to as major complications.

### Statistical analysis

SPSS (IBM SPSS Statistics for Windows, Version 21.0. Armonk, NY: IBM Corp) was used for statistical analysis.

All variables are shown as median and range or with 95% confidence interval (CI). Variables were compared between the subgroups using the Mann-Whitney U test for two subgroups. Correlations were calculated using the Pearson rank correlation test. Survival analysis was performed using a Kaplan–Meier estimator. For comparison, the log-rank test was used. For odds ratio (OR) calculation 2 × 2 crosstabs were used. *P*-values < 0.05 were considered significant. Sample size was calculated using published morbidity rates by Luketich et al., as well as morbidity of our own pilot trial [12,14]. For demonstration of statistical difference, two groups of 20 patients each were calculated using an α of 0.05 and β of 0.80.

## Results

### Patients

From 5/2010 to 12/2012, 76 patients underwent esophageal resection at the Department of Surgery of the Medical University of Vienna. In that period, 29 patients were eligible for participation in the MIOMIE study. Three (11.5%) patients dropped out due to progression of disease and merely underwent explorative surgery. Finally, 26 (20 male, 76.9%) patients underwent surgery after randomization. The median age was 63 years (40–77) and 21 (80.8%) patients were treated for AC, whereas only 5 (19.2%) patients were operated on for ESCC. Sixteen (61.5%) patients received neoadjuvant treatment as part of a multimodal treatment strategy. There was no difference of median BMI in the OE group (26.96 kg/m^2^, 17.53–35.26) to the MIE group (24.08 kg/m^2^, 18.07–41.45; *p* = 0.556). Summary of demographic data is shown in Table 1.

**Table 1:** Demographics and tumor-related details

Variable	All (*n* = 26)	OE (*n* = 12)	MIE (*n* = 14)	*P-*value^b^
Median age, years^a^	63 (40–77)	62.5 (49–77)	64.5 (40–75)	–
*Gender*				
Male	20 (76.9)	10 (83.3)	10 (71.4)	0.473
Female	6 (23.1)	2 (16.7)	4 (28.6)	
Body mass index, kg/m^2a^	25.73 (17.53–41.45)	26.96 (17.53–35.26)	24.08 (18.07–41.45)	0.556^c^
History of tobacco use	12 (46.2)	5 (42.7)	7 (50.0)	0.671
Stent bridging	3 (11.5)	1 (8.3)	2 (14.3)	0.636
*Tumor location*				
Thoracic	5 (19.2)	1 (8.3)	4 (28.6)	0.264
Siewert type I	20 (76.9)	10 (82.3)	10 (71.4)	–
Siewert type II	1 (3.8)	1 (8.3)	–	–
Siewert type III	–	–	–	–
*Tumor histology*				
Adenocarcinoma	21 (80.8)	11 (91.7)	10 (71.4)	0.192
Squamous cell carcinoma	5 (19.2)	1 (8.3)	4 (28.6)	–
*Neoadjuvant treatment*				
Chemotherapy	16 (61.5)	7 (58.3)	9 (64.3)	0.307
*Neoadjuvant regimen*				
Taxane-based	8 (30.8)	5 (42.7)	3 (21.4)	–
Platinum-based	8 (30.8)	2 (16.7)	6 (42.9)	–
*Tumor grading*				
Well differentiated (G1)	1 (3.8)	–	1 (7.1)	0.413
Moderately differentiated (G2)	16 (61.5)	6 (50)	10 (71.4)	–
Poorly differentiated (G3)	6 (23.1)	4 (33.3)	2 (14.3)	–
No grading possible (Gx)	3 (11.5)	2 (16.7)	1 (7.1)	–
*Pathologic tumor stage*				
T1	8 (30.8)	4 (33.3)	4 (28.6)	0.880
T2	4 (15.4)	2 (16.7)	2 (14.3)	–
T3	9 (34.6)	3 (25.0)	6 (42.9)	–
T4a	2 (7.7)	1 (8.3)	1 (7.1)	–
T0	3 (11.5)	2 (16.7)	1 (7.1)	–
*Pathologic nodal stage*				
N0	14 (53.8)	7 (58.3)	7 (50.0)	0.224
N1	8 (30.8)	3 (25.0)	5 (35.7)	–
N2	2 (7.7)	2 (16.7)	–	–
N3	2 (7.7)	–	2 (14.3)	–
*Surgical margin status*				
Clear	24 (92.3)	12 (100.0)	12 (85.7)	0.395
Microscopically involved (R1)	1 (3.8)	–	1 (7.1)	–
Macroscopically involved (R2)	1 (3.8)	–	1 (7.1)	–

Values in parentheses are percentages unless indicated otherwise

*OE* open esophagectomy, *MIE* minimal invasive esophagectomy

^a^values are median (range)

^b^χ^2^ test

^c^independent samples t‑test

### Surgery

Open surgery was performed in 12 (46.2%) patients and 14 (53.8%) patients underwent laparoscopic gastric tube formation. In one (7.1%) patient, laparoscopy had to be converted to an open access due to technical difficulties. In one patient of each group, a small atypical lung resection was performed due to suspicious findings.

The median operation time in the OE group was 290 (215–385) minutes and 292.5 (200–450) minutes in the MIE group. The abdominal surgerical part showed a trend towards a reduced operation time in the OE group. Nevertheless it failed to reach statistical significance: OE: 155 (120–240) minutes versus MIE: 175 (110–255) minutes (*p* = 0.06). Operation time in the thoracic part remained equal, as expected: OE 132.5 (80–145) minutes versus MIE 117.5 (90–196) minutes.

Pyloromyotomy or pyloroplasty was not performed in the MIE group, but dilatation of the pylorus was carried out in the OE group. Further details are summarized in Table 2.

**Table 2:** Perioperative details

Variable	All (*n* = 26)	OE (*n* = 12)	MIE (*n* = 14)	*P*-value^a^
Length of operation	290 (200–450)	290 (215–385)	292.5 (200–450)	0.421
Abdominal part, median	170 (110–255)	155 (120–240)	175 (110–255)	0.060
Thoracic part, median	120 (80–95)	132.5 (80–145)	117.5 (90–195)	0.899
Duration of ventilation (days)	0 (0–18)	0 (0–18)	0 (0–17)	0.940
ICU stay (days)	4 (1–69)	4 (1–44)	5 (3–69)	0.297
Hospital stay (days)	12.5 (7–77)	13 (9–44)	14 (7–77)	0.940

Values in parentheses are ranges

*ICU* intensive care unit

^a^skewed distribution, Mann–Whitney test applied

### Morbidity

Minor and major complications occurred in 2 (7.7%) and 10 (38.5%) patients, respectively. Anastomotic leakage (AL) was seen in 2 (16.6%) patients in the OE group and in 3 (21.4%) cases in the MIE group (OR 1.364; CI = 0.188–9.912; *p* = 0.759). Summing up, there was no significant difference between the OE and the MIE group regarding surgical (AL, gastric conduit necrosis, reoperation rate, C/D classification) or pulmonary complications. One patient died in the OE group due to pulmonary complications (acute respiratory distress syndrome following pneumonia and pulmonary embolism). For further details, please see Table 3.

**Table 3:** Morbidity in OE versus MIE

Variable	All (*n* = 26)	OE (*n* = 12)	MIE (*n* = 14)	OR (95%CI)	*P*-value^a^
C/D I & II	2 (7.7)	1 (8.3)	1 (7.1)	0.385 (0.300–4.867)	0.722
C/D IIIa,b & IVa,b	10 (38.5)	4 (33.3)	6 (35.7)	1.714 (0.339–8.676)	–
C/D V	1 (3.8)	1 (8.3)	0	–	–
Anastomotic leakage	5 (19.2)	2 (16.6)	3 (21.4)	1.364 (0.188–9.912)	0.759
Gastric conduit necrosis	1 (3.8)	0	1 (7.1)	–	0.345
Reoperation	8 (30.8)	4 (33.3)	4 (28.6)	0.800 (0.151–4.245)	0.793
Pulmonary complication	6 (23.1)	3 (25)	3 (21.4)	0.818 (0.132–5.084)	0.271

Values in parentheses are percentages, except OR column

*C/D *Clavien/Dindo grade, *OE* open esophagectomy, *MIE* minimal invasive esophagectomy

^a^χ^2^ test

### Long-term outcome

**Fig. 2:**
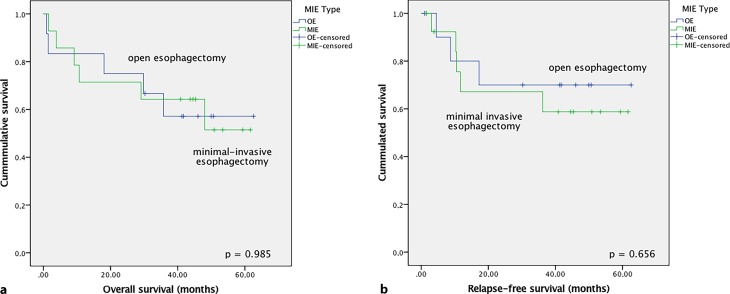
Kaplan–Meier analysis of overall (**a**) and relapse-free survival (**b**) of patients treated with open esophagectomy (OE) vs. minimally invasive esophagectomy (MIE)

With a median follow-up of 41.5 (1–62.6) months, the overall 2‑year survival rate was 61% and the 5‑year survival rate was 50%. Eight (30.8%) patients developed recurrence of disease in the study period. There was no significant difference in OS and RFS regarding type of surgery (*p* = 0.985 and *p* = 0.656; Fig. 2).

## Discussion

Herein, we present our single-center randomized controlled trial where we could demonstrate that morbidity and mortality were similar between minimally invasive and open procedures.

After MIE was proven to be a feasible and safe procedure over the past decade, we introduced this technique at our institution [4,15,16]. In a case–control study of the first 62 MIE patients, we found that implementation of this approach caused no difficulties [12]. The academic consequence of this analysis was the design of a prospective randomized trial to minimize selection bias and specific limitations of a retrospective study.

Goal of this RCT was to prove equal morbidity and furthermore similar oncological results of MIE compared to OE. With respect to a distinct study design, we chose patients with a comparable nature of disease who could be treated with Ivor Lewis esophagectomy. In order to not compromise oncological benefits, we decided to perform anterolateral minithoracotomy and intrathoracic anastomosis in all cases, as described by Briez and colleagues in the MIRO trial [17].

The MIOMIE study was primarily designed with 20 patients in each group. After 10 patients each, we saw an alarming incidence of consecutive AL (19.2%), which we linked to an issue with a stapling device. Thus, we decided to end the RCT, to not adulterate further results by adapting inevitable technical steps. Now presenting the long-term results of an interrupted RCT, we show that MIE and OE can be equally performed, and results regarding morbidity and also oncological outcome are comparable and reproducible.

Despite the increased AL rate (19.2%), the major complication rate was 38.5%. This is similar compared to other studies, but unlike Briez et al. in 2012, we did not find any difference between the groups (MIE 35.7% vs. OE 33.3%) [18]. Also, unlike the presented results of the MIRO trial: the rate of major complications in our MIE group was similar to Mariette’s results in the large multicenter setting, but we did not find an increased morbidity rate in the OE group (MIRO trial: 64.4%) [19]. The recent publication of a large multicenter series of Luketich indicated an AL rate of 8.6% [8]. The study setting was different though, and only patients treated with laparoscopy and VATS were included. The low AL rate Luketich reported can be seen as a benchmark for both the MIE and the OE technique. In contrast to our earlier publication from 2010, the complication rate in the MIE group remained stable, whereas the total complication rate in the OE group dropped from initially 74.2 to 41.7%. Going into detail, we can see that the major complication rate improved less than minor complications (major: 38.7 to 33.3% vs. minor: 41.9 to 8.3%). This finding is mainly due to the fact that we did not do cervical resections and have improved the perioperative setting in OE since then. Moreover, the lacking thoracoscopic approach may be another explanation. In the previous analysis, we saw a reduced rate of respiratory insufficiency in the thoracoscopic group [12].

Furthermore, our results demonstrate that MIE does not impact on oncological outcome. We found a 2-year survival rate of 61%, which is in line with the survival rate of Luketich et al. in the E2202 study (2-year survival rate of 68%). With a 5-year survival of 50%, we provide oncological long-term results of a prospective study [8].

When presenting data of an interrupted RCT, certain limitations should be stated. Data interpretation, especially regarding long-term survival, has to be done carefully due to low treatment numbers. Our primary goal of 20 patients in each group was only fulfilled to 50%. Learning curve cannot be assessed, as MIE was established as a standard procedure at our institution. This is also displayed by a similar operation duration in both groups.

However, performing a prospective series after establishing a new surgical approach needs to be done as a consequence of the first analysis. Although, interrupting this trial due to safety reasons restricts statistical interpretation. Still, we decided to publish our experience to avoid a possible publication bias and encourage the design of further studies regarding this matter [20].

## Conclusion

This study shows that MIE is a feasible option for esophageal resection. Morbidity, mortality, and oncological long-term outcome were equal to the classical open Ivor Lewis approach.

In contrast to other studies, however, we did not find a reduced morbidity in MIE. Therefore, decision for the minimally invasive approach still remains with the surgeon’s point of view and his specific experience.
